# The Longitudinal Link between Organizational Citizenship Behaviors and Three Different Models of Happiness

**DOI:** 10.3390/ijerph18126387

**Published:** 2021-06-12

**Authors:** Wenceslao Unanue, Eduardo Barros, Marcos Gómez

**Affiliations:** School of Business, Universidad Adolfo Ibáñez, 7910000 Peñalolén, Chile; eduardo.barros@uai.cl (E.B.); marcos.gomez@uai.cl (M.G.)

**Keywords:** organizational citizenship behaviors, hedonic happiness, eudaimonic happiness, flourishing, longitudinal analysis, prospective design

## Abstract

A growing body of research conducted in general life settings has found positive associations between happiness and prosocial behavior. Unfortunately, equivalent studies in the workplace are lacking. Organizational citizenship behaviors (OCBs), the prosocial behaviors at work, have not been properly studied in relation to happiness, despite the positive consequences of both constructs for workers and companies. In response, our research aims to better understand this relationship from several angles. First, using a three-wave longitudinal design, we explored how OCBs and happiness are related to each other over time. Second, happiness was measured from a broad perspective, and three conceptualizations were adopted: the hedonic (e.g., positive affect and life satisfaction), the eudaimonic (e.g., relatedness and autonomy), and the flourishing (e.g., meaning and engagement) approaches. Thus, not only the prospective link between OCBs and happiness was tested, but it was also explored using the three models of happiness previously mentioned. Third, we conducted this longitudinal design in a less typical sample than previous research (i.e., Chile). We found results that supported our main hypotheses: (1) OCBs are prospective positive predictors of hedonic happiness, eudaimonic happiness, and flourishing; (2) the three models of happiness also prospectively predict OCBs. Our findings suggest that OCBs foster a broad range of happiness facets, which in turn fosters back the emergence of more OCBs, leading to a virtuous circle of prosociality and well-being in the workplace. This positive spiral benefits not only workers’ quality of life, but also organizations’ profitability and sustainability. Theoretical and applied implications for the field of Positive Organizational Psychology are discussed.

## 1. Introduction

“The best way to find your own happiness and the happiness of others, is to open your heart … so be good, do good.”

Matthieu Ricard

A growing body of research conducted in general life settings has found positive associations between happiness and prosocial behaviors (PSBs; see [1,2,3,4]). Unfortunately, the equivalent of PSBs in the workplace, organizational citizenship behaviors (OCBs), have not been studied in the same manner in relation to happiness. Since research has consistently shown the positive consequences of happiness and OCBs for both workers and organizations (e.g., enhanced health, job satisfaction, performance, etc.; see [5,6,7,8,9]), studying the particular features of this relationship is worthy.

Aiming to fill the previous research gap, we explored prospectively the link between OCBs and happiness. In addition, we studied happiness from a holistic perspective, using three well-known conceptualizations of happiness: the hedonic approach, the eudaimonic approach, and the flourishing approach [10,11,12]. Each of the three happiness conceptualizations may provide unique and important information regarding the happiness-OCBs link in the study.

Drawing on a substantial body of research in psychology, we theorized that the OCBs–happiness link is reciprocal and that this bi-directionality applies to these three happiness conceptualizations. Indeed, despite the fact that most research on well-being and OCBs presumes that well-being is the driver of OCBs [13], both well-being (e.g., [14]) and organizational researchers (e.g., [15]) state that the opposite direction may also be possible (see, [16]). Further, we conducted a three-wave longitudinal design, using a cross-lagged panel model (CLPM). The CLPM conducted allowed us to test prospective (i.e., temporal) directions between OCBs and happiness [17]. Although CLPMs do not test causality directly, prospective significance between constructs is a key requirement for causality. Indeed, CLPMs allow “looking at autoregressive effects (linking a variable at earlier time points to itself at later time points) and cross-lagged effects (linking two different variables across time)” [18] (p. 183), [19]. Therefore, our design would not only allow testing the associations between the constructs of interest, but also disentangling the right directionality between them.

To our best knowledge, this research is the first to date to explore the reciprocal longitudinal link between OCBs and three conceptualizations of happiness in an organizational context. Further, we expect that our results bring new insights to academics and practitioners in the Positive Organization Psychology field (POS), which aims to improve workers’ quality of life as well as build healthier and more resilient organizations [20]. We hope that our findings help POS to advance its knowledge regarding how employees and organizations may increase their performance and, at the same time, flourish.

## 2. Theoretical Background

### 2.1. Happiness

Happiness is a broad construct that has been conceptualized from three psychological perspectives: the hedonic, eudaimonic, and flourishing approaches [21,22]. In this section, we will characterize these three conceptualizations.

#### 2.1.1. Hedonic Happiness

Hedonic theories have formed the most extensively studied approach to happiness [7,23]. This view defines happiness in terms of attaining pleasure and avoiding pain [11]. The construct of subjective well-being (SWB) was developed by Diener [24] to measure hedonic happiness. SWB assesses people’s sense of wellness, in both thoughts and feelings, and includes three facets: satisfaction with life, positive affect, and negative affect [8]. Life satisfaction, the cognitive aspect of SWB, captures satisfaction with one’s life in a broad sense (e.g., marriage, job, health). Positive (e.g., pleasure, enjoyment, contentment, love) and negative (e.g., fear, anger, and depression) feelings capture the emotional aspects of SWB. Further, in this theorization, a happy person thinks her/his life is going well and experiences frequent positive affect and only occasional negative affect [8].

#### 2.1.2. Eudaimonic Happiness

According to the eudaimonic perspective, happiness is more than feeling pleasure and avoiding pain. Happiness reflects the actualization of human potential, meaning, and self-realization [10,11,22]. Further, eudaimonic approaches conceptualize happiness in terms of being fully functioning and having a life that is worth living [11,23,25]. Basic psychological needs theory (BPNT; [26,27]), one of the six self-determination theory (SDT) “mini-theories” [28], is a theory of eudaimonic happiness [10]. Following Deci and Ryan’s [26] original ideas, just as plants need essential nutrients—such as water, sunlight, and minerals—for survival, so people need psychological nutrients for healthy growth and well-being [29]. BPNT states that the satisfaction of the three basic psychological needs (BPNS)—autonomy, competence, and relatedness—would function as the necessary psychological nutrients for wellness and optimal functioning. Autonomy refers to the feeling that our behavior is volitional and meaningful; competence refers to feeling effective and efficient in our behavior, as well as being able to successfully manage difficult challenges; relatedness refers to feeling that we are connected, appreciated, and understood by others who are important to us through intimate relationships [28,30,31,32].

#### 2.1.3. Flourishing

Although the hedonic and eudaimonic approaches have evolved separately, attention is increasingly being given to how both perspectives are connected [25]. Indeed, focusing only on hedonic or eudaimonic aspects of happiness has been strongly questioned [22,33], resulting “in the development of more integrated frameworks” [10] (p. 186). Flourishing is one of the terms that several academics have used to unify the eudaimonic and hedonic models of happiness [12,34,35]. The PERMA model is the most popular conceptualization of flourishing. It was developed by Martin Seligman, one of the founders of the Positive Psychology field [36]. Seligman [12] proposed a theory of well-being, in which flourishing is defined in terms of five pillars: positive emotions (P), engagement (E), relationships (R), meaning (M), and accomplishment (A). To disseminate his theory, the author published a book called, “Flourish: A visionary new understanding of happiness and well-being.” Seligman’s book has sold more than 70,000 copies worldwide, inspiring a multiplicity of researchers, practitioners, conferences, and governments around the world (see, [37]). Despite its vast popularity and influence in the positive psychology field, research using the PERMA model is scant, and only a few studies have been published to date (see, [38,39,40]). We think that the PERMA model is a promising avenue for predicting quality of life, but the evidence is still in progress. Further, by including it in our research, we are contributing to overcoming the scarce literature using this framework.

We decided to explore the three above-mentioned psychological perspectives of happiness in our paper for two main reasons. First, understanding happiness only from the hedonic perspective may lead people to think, misleadingly, that humans only search for pleasure and avoid pain. Yet, people are more complex entities. Beyond pleasure and pain, humans need, for example, to build strong social ties and find meaning in life. These facets are captured by the eudaimonic and flourishing models, respectively. Second, each of the three happiness conceptualizations may provide unique and important information regarding the happiness–OCBs link in the study.

### 2.2. Organizational Citizenship Behaviors

OCB, a kind of pro-social behavior conducted within the workplace, refers to “individual behavior that is discretionary, not directly or explicitly recognized by the formal reward system, and in the aggregate promotes the efficient and effective functioning of the organization” [41] (p. 6). In other words, OCBs are behaviors that are not specified in the job description and involve personal choice, and the workers are not punished for engaging or not in the act [41].

For many decades, OCBs have been conceptualized through different approaches [5,42]. The roots of OCBs are attributed to Katz [43], who used the term innovative and spontaneous behaviors. The author stated that these behaviors comprise five key dimensions: cooperation acts, protection acts, constructive ideas, self-training, and favorable attitudes. Organ [44] and Smith et al. [45] were the first to propose the term OCBs. Smith et al. [45] argued that facets such as acts of cooperation, helpfulness, suggestions, gestures of goodwill, and altruism may be considered citizenship behaviors. The authors also suggested two dimensions: altruism (actions of helping toward individuals) and general compliance (prosocial behaviors directed towards the organization). Similarly, Williams and Anderson [46] suggested classifying OCBs into two dimensions: OCBs-I (prosocial behaviors towards individuals) and OCBs-O (prosocial behaviors towards the organization).

Podsakoff et al.’s [47] meta-analysis concluded that the most cited dimensions of OCBs were helping behavior, sportsmanship, organizational loyalty, organizational compliance, individual initiative, civic virtue, and self-development. Interestingly, most dimensions found by Podsakoff et al. [47] collapsed with the facets proposed by Katz [47]. LePine et al. [48] supported previous findings. The authors reviewed the literature on OCBs and their dimensions in order to test the widely held assumption that the behavioral dimensions of OCBs are distinct from one another. Using meta-analytic techniques, they found “strong relationships among most of the dimensions and that the dimensions have equivalent relationships with the predictors” (p. 52).

Thus, despite a multiplicity of dimensions that have emerged over decades to characterize OCBs [5], there is strong agreement that OCBs are acts of helping, kindness, and generosity toward individuals and organizations. As an analogy, if civic citizenship behavior is the “exemplar” behavior of every citizen, OCB is the “exemplar” behavior of every worker [49].

### 2.3. Research Gaps for the Link between OCBs and Happiness

Prosocial behaviors (e.g., donating blood, volunteering, giving time, and money; PSBs) “represent a broad category of acts that are defined by some significant segment of society and/or one’s social group as generally beneficial to other people” [50] (p. 366). Research has found positive associations between happiness and PSBs in general life settings [3,4,51,52,53]. Recent studies have even suggested that the link may be causal and reciprocal: People who engage more in PSBs are happier, but also happier people tend to engage more in PSBs [1,2]. Unfortunately, this kind of research is lacking in the workplace, and the link between OCBs (prosocial acts in organizations) and happiness has not been tested yet. Further, we identified three main research gaps.

First, as far as we know, just a few studies conducted in the workplace have explored the link between OCBs and only some facets of hedonic (e.g., positive affect; [13,54]) and eudaimonic happiness (e.g., autonomy; [55]). Unfortunately, no research to date has explored the link between OCBs and any proper models/construct of happiness. Second, the directionality of the link is not clear yet. Indeed, no research to date has explored how both constructs relate to each other over time. Do OCBs lead to higher happiness or does happiness lead to higher OCBs? Is it possible to have a reciprocal link? These questions may be solved using longitudinal designs. However, for quite a long time, researchers have been claiming the lack of longitudinal research in this field of study [56]. Third, the scarcity of studies exploring OCBs and some facets of happiness are culturally laden. Indeed, there is a lack of cultural diversity. Previous attempts to explore the link between OCBs and specific facets of happiness have been conducted mainly in the Western World in countries such as the US (e.g., [13,16]) and Canada [57,58], with a few exceptions in China [59,60] and Indonesia [61].

In the same vein, Urbach et al. [62] state that “the effect of societal culture and its associated values has received little research attention to date” (p. 2). That is unfortunate, because cultural differences may affect both the antecedents and the consequences of employees’ proactive work behaviors (such as some OCBs). Thus, testing the link between OCBs and happiness in different cultural contexts seems needed. Chile, a Latin American country going through a fast economic and social transition, is different from the North American and Asian countries usually included in this field of research (see Unanue et al. [19] for details). Therefore, we decided to test our hypotheses in a Chilean sample, in order to go beyond the East–West dichotomy [63]. Our research aims to fill previous research gaps.

## 3. Hypotheses Development

### 3.1. The Hypothesized Reciprocal Link between SWB and OCBs

#### 3.1.1. The Link from SWB to OCBs

We used the SWB construct [24] to measure hedonic happiness. People high in SWB have a “sense of wellness in their lives—in both thoughts and feelings”. That is because individuals with high SWB tend to show high levels of life satisfaction and positive affect, as well as low levels of negative affect [8] (p. 90). Therefore, feeling and thinking that our lives are going well lead us to be more inclusive and sympathetic to other people. In the workplace, happiness should have similar consequences. Indeed, we expect that workers high in SWB tend to see their colleagues and organizations in a more positive way, feel greater compassion, have more sympathy, and desire to help [8], which in turn, could promote the pursuit of OCBs [64]. In other words, high SWB leads employees to perceive things in a more positive light, which consecutively increases the likelihood of feeling positive towards co-workers, encouraging helpfulness, and thus OCBs [65]. Because high SWB is self-reinforcing, when people feel good at work, employees try to maintain these positive feelings through, for instance, protecting the organization with more OCBs. By protecting the organization, “the worker in a positive mood helps to ensure that his or her own good mood is maintained” [65] (p. 316). Moreover, positive moods also foster creativity and innovation, thus leading workers to make potentially more constructive suggestions for the organizations. People high in SWB are also more self-confident, self-efficacious, and capable at work, leading them to increase their aspirations and get involved in more self-development activities, which bring benefits not only to themselves, but also to the whole organizational environment. Finally, workers high in SWB “are more likely to evaluate the organization favorably and, hence, spread goodwill” [65] (p. 317). Hence, we have the following hypothesis.

**Hypothesis 1a** **(H1a).** *Hedonic happiness (i.e., SWB) prospectively predicts future OCBs.*

#### 3.1.2. The Link from OCBs to SWB

OCBs increase SWB, helping workers to feel and think that their lives are satisfactory and rewarding, to experience more positive and less negative feelings, and have better life evaluations. One of the theoretical explanations for the impact of OCBs on SWB comes from novel studies on benevolence. Recent research has found that OCBs predict higher benevolence (a sense of having a positive impact on others; Gomez et al. [66]), which in turn increases SWB [52]. Additional support for the link from OCBs to SWB comes from van der Linden’s (2012) Helper’s theory (cited in [67]). The author suggests that any kind of prosocial behavior (i.e., OCBs) leads to the “feel-good” effect through the activation of neurotransmitters that fosters positive feelings and cognitions in the giver. Additionally, by experiencing positive emotions, there is less room left for negative emotions, which also increases SWB. Therefore, we have the following hypothesis:

**Hypothesis 1b** **(H1b).** *OCBs prospectively predict future hedonic happiness (i.e., SWB).*

### 3.2. The Hypothesized Reciprocal Link between BPNS and OCBs

#### 3.2.1. The Link from BPNS to OCBs

We used the SDT framework [26,28] to conceptualize eudaimonic happiness through the satisfaction of the basic psychological needs for autonomy, competence, and relatedness (BPNS; [26,27]). SDT claims that “we are naturally inclined to be prosocial animals, given proper nurturing” [68] (p. 202). When our needs for autonomy, competence, and relatedness are fulfilled, we have more vital energy and, thus, we are more likely to engage in behaviors such as helping colleagues at work [68]. Furthermore, workers who have their needs met, are more likely to get involved in extra-role behaviors (a key feature of OCBs). For example, the satisfaction of the need for autonomy could energize workers into helping colleagues and the organization, “both as a response to social exchange obligations, but perhaps also through having greater time to spend on activities beyond their immediate job requirements” [69] (p. 4). Actually, the concept of autonomy refers to the power and freedom felt by employees when organizing their job activities according to their own pace and priorities. Thus, the higher the autonomy, the higher the opportunities to show voluntarily extra-role behavior such as OCBs. Autonomy is a motivational force that leads employees to put extra effort into OCBs, going beyond the formal work requirements [55,70].

The satisfaction of the need for competence (as a result of their enhanced efficiency and effectiveness) may also give employees enough energy as well as confidence in their abilities [59], leading them to give greater assistance to colleagues and the organization [71]. Additionally, competence satisfaction may predict higher OCBs, because demonstrating and improving one’s abilities through these kinds of extra-role behaviors is fundamentally satisfying [72]. The satisfaction of the need for relatedness helps employees to feel connected to and cared for by relevant others at work, which consecutively leads them to feel an increased sense of belonging and increased motivation to help those around them [60]. Indeed, “a satisfied sense of relatedness provides a secure base of support encouraging novel, exploratory, and potentially risky endeavors” such as creative and prosocial behaviors” [60] (p. 1041). Thus, higher relatedness provides a background that enables employees to display initiative in extra-role behaviors. Importantly, the needs for relatedness “include the desire to connect with others, to give affection, and to receive love and care in return” [72] (p. 874). Thus, in order to satisfy this need, workers may establish emotional bonds through OCBs.

To summarize, higher BPNS fosters interest in supporting one’s organization and colleagues through higher levels of OCBs [60]. Indeed, BPNS may increase the likelihood of workers practicing OCBs, because they represent an energetic resource that propels a variety of self-motivated behaviors [73,74]. Therefore, we have the following hypothesis:

**Hypothesis 2a** **(H2a).** *Eudaimonic happiness (i.e., BPNS) prospectively predicts future OCBs.*

#### 3.2.2. The Link from OCBs to BPNS

Drawing on SDT research (SDT; [28]), we hypothesized that PSBs (i.e., OCBs) foster the satisfaction of the basic psychological needs for autonomy, competence, and relatedness [53]. OCBs might boost the satisfaction of the need for autonomy, because the volitional act of helping (a core characteristic of OCBs) “can be experienced as truly self-initiated and endorsed”, promoting the perception of autonomy [53] (p. 224). OCBs also enhance the psychological need for competence, because workers may feel that they are producing positive changes in the lives of the recipients of their help. Indeed, extra-role behaviors such as helping others “elicit experiences of competence, involvement, and usefulness” [53] (p. 224) as well as self-efficacy.

Finally, OCBs promote the satisfaction of the need for relatedness. Actually, OCBs are “inherently interpersonal and thus impact relatedness by directly promoting closeness to others, positive responses from others, and cohesiveness or intimacy” [53] (p. 224). Thus, in the workplace, OCBs are key nutrients for keeping meaningful relationships with workers and the organization. To summarize, OCBs may foster BPNS, because OCBs are associated with higher levels of the three needs. OCBs may satisfy the need for autonomy, “insofar as prosocial acts are volitional and autonomous”. OCBs may also satisfy the need for competence, “insofar as one feels effective in helping”. Finally, OCBs may also satisfy the need for relatedness, “insofar as one feels more connected with others” [51] (p.751). Therefore, we have the following hypothesis:

**Hypothesis 2b** **(H2b).** *OCBs prospectively predict future eudaimonic happiness (i.e., BPNS).*

### 3.3. The Hypothesized Reciprocal Link between PERMA and OCBs

We used the PERMA model of well-being to conceptualize flourishing (Seligman, 2011). Since Seligman includes both hedonic and eudaimonic components of happiness in the PERMA model, and we have already extensively explained the psychological mechanisms at the basis of a potential bi-directional link between these two conceptualizations of happiness and OCBs, we also expect a similar relation of PERMA with OCBs. Hence, we have the following hypotheses:

**Hypothesis 3a** **(H3a).** *Flourishing (i.e., the PERMA model) prospectively predicts future OCBs.*

**Hypothesis 3b** **(H3b).** *OCBs prospectively predict future flourishing (i.e., the PERMA model).*

## 4. The Present Research

In the previous sections, we have identified several research gaps regarding the study of the OCB–happiness link. Therefore, the main goal of our study is to fill these gaps by exploring prospectively the link between OCBs and the three models of happiness already explained (hedonic happiness, eudaimonic happiness, and flourishing). To the best of our knowledge, this is the first longitudinal study to date to test not only the hypothesized link from happiness to OCBs, but also the hypothesized reverse link from OCBs to happiness in an organizational context. In addition, we extended previous research conducted mainly in a small number of countries in the Western world, with a few exceptions in Asia. We analyzed data from Chile, going beyond the traditional Western–Eastern paradox [63].

## 5. Method

### 5.1. Procedure

Our research is part of a large longitudinal project on happiness and well-being funded by the Chilean government. It was approved by the Ethics and Research Committee from a Chilean university and followed ethical procedures to avoid coercion (e.g., participation was voluntary and online; no penalties were applied in case of leaving the study). At T1, Chilean workers received an online invitation to participate in a three-wave longitudinal study and were asked to consent to future waves (T2 and T3). Consenting T1 participants were sent an email containing a web-link to the questionnaire. Participants who finished the T1 survey were invited to participate at T2 and T3. Those participants who decided not to participate in the study (at any time/wave) were given the option to unsubscribe from the mailing list and were not contacted later. In each wave, respondents were notified that the survey would be available for only one week. Kind reminders were sent twice in each wave.

### 5.2. Sample

Data for our core variables were obtained from a three-wave longitudinal survey with one month between each wave (T1 *n* = 735 (42.97% female; Mean age = 38.77; *SD* = 9.69); T2 *n* = 282; T3 *n* = 288; for more details see Table 1). Regarding attrition, those who completed our main constructs in all the three waves (*n* = 171, 23%) did not differ significantly in age, gender, SWB, BPNS, PERMA, or OCBs from those who left after T1 or T2 (*n* = 564; *p* ≥ 0.04). A total of 15% of 735 participated in T1 and T2, 16% participated in T1 and T3, and 46% participated in T1 only. The results of the Little MCAR test [75] showed that missing data were completely at random for the three models (Model 1: χ^2^(126) = 152.88, *p* = 0.052; Model 2: χ^2^(108) = 128.86, *p* = 0.084; Model 3: χ^2^(144) = 166.78, *p* = 0.094). Thus, we included all 735 participants in our structural analyses, using FIML to handle missing data [76]. FIML infers the model log-likelihoods using all observed cases, obtaining equivalent results that of multiple imputation methods if the data missing mechanism is MCAR or MAR [77]. Additionally, we compared the obtained estimates to their listwise counterpart, and because the observed data were MAR, the results were very similar. The estimates’ raw differences ranged from 0.00 to 0.04, showing the same pattern of results, yet reaching non-significance levels for Model 1 and 3 due to sample loss, while results from Model 2 supported the same conclusions.

Regarding normality, the assumption is tenable if skewness ranges from −2 to +2 and kurtosis ranges from −7 to +7 [78,79,80]; all skewness (−1.74 to −0.14) and kurtosis values (2.69 to 7.04) ranged acceptably.

Recommended sample sizes for SEM vary widely. Wolf et al. [81] have shown that as many as 460 participants may be sufficient and that the required sample size does not necessarily increase with model complexity. Thus, we judge that our current sample size (*N* = 735) is good enough for testing all our longitudinal SEM models.

### 5.3. Measures

All constructs were captured through highly validated scales, which were translated into Spanish using a standard back-translation procedure [82].

#### 5.3.1. Hedonic Happiness

We used the SWB construct developed by Diener [24]. SWB consisted of three sub-scales: life satisfaction, positive affect, and negative affect.

Positive and Negative Affect. We used the 10-item International Positive and Negative Affect Schedule Short Form (I-PANAS-SF; [83]) to measure positive (5 items) and negative affect (5 items). Example items asked participants how frequently they had felt “inspired”, “alert”, “upset”, or “nervous” during the last month, ranging from never (1) to always (5). Cronbach’s alphas for positive affect were good at T1 (0.72), T2 (0.74), and T3 (0.73). Cronbach’s alphas for negative affect were acceptable at T1 (0.69), T2 (0.74), and T3 (0.75).

Life Satisfaction. We used the 5-item Satisfaction with Life Scale [84]. Participants rated five items, such as “in most ways my life is close to my ideal” and “the conditions of my life are excellent” on a 7-point Likert-type scale ranging from strongly disagree to strongly agree. Cronbach’s alphas were good at T1 (0.91), T2 (0.93), and T3 (0.93). We built a latent variable for SWB. We created five item parcels as indicators, using domain-representative parcels [85]. This parcel construction consisted of including a single parcel item from each scale facet (one positive affect, one negative affect, and one life satisfaction item). This parcel strategy aimed to maximize SWB variance while canceling out nuisance factors in the interest of the study. To summarize, each SWB latent variable was built with 5 parcels as indicators. Each of the 5 parcels has 3 items.

#### 5.3.2. Eudaimonic Happiness

We used the BPNS scale, a 12-item scale developed by Chen et al. [86]. Participants rated from 1 (not at all true) to 7 (very true) their satisfaction of the needs for autonomy, competence, and relatedness. Example items (four for each need) included “I feel my choices express who I really am” (autonomy), “I feel capable at what I do” (competence), and “I feel connected with people who care for me, and for whom I care” (relatedness). Cronbach’s alphas were good at T1 (0.90), T2 (0.93), and T3 (0.94). We built latent variables for BPNS. We used four item parcels as indicators of each latent variable. To ensure that each latent variable would equally represent all three needs, each parcel used one autonomy, one competence, and one relatedness item. To summarize, each BPNS latent variable was built with 4 parcels as indicators. Each of the 4 parcels has 3 items.

#### 5.3.3. Flourishing

We used the PERMA-Profiler [87]. The measure captures the five components proposed by Seligman [12], with three questions per construct. Participants answered on an 11-point scale ranging from 0 to 10, and the items within each construct were averaged together to create an indicator of that domain. Example items are “In general, how often do you feel joyful?” (P: positive emotions); “In general, to what extent do you feel excited and interested in things?” (E: engagement); “To what extent have you been feeling loved?” (R: relationships); “In general, to what extent do you feel that what you do in your life is valuable and worthwhile?” (M: meaning); and “How often do you achieve the important goals you have set for yourself?” (A: accomplishment). Cronbach’s alphas for the PERMA scale were good at T1 (0.93), T2 (0.95), and T3 (0.96). We built latent variables for flourishing. We used three item parcels as indicators. To ensure that each latent variable would equally represent all five dimensions, each parcel used one positive emotion, one engagement, one relationship, one meaning, and one accomplishment item. To summarize, each PERMA latent variable was built with 3 parcels as indicators. Each of the 3 parcels has 5 items.

#### 5.3.4. Organizational Citizenship Behaviors

We used the 16-item scale developed by Lee and Allen [57]. Participants answered on a 7-point scale from 0 (never) to 7 (always) how often they behave in several ways. Example items are “help others who have been absent” and “attend functions that are not required but that help the organizational image”. Cronbach’s alphas for the OCBs scale were good at T1 (0.92), T2 (0.93), and T3 (0.95). We built latent variables for OCBs. We used three item parcels as indicators, balancing the 16 scale items. To summarize, each OCB latent variable was built with 3 parcels as indicators. Whereas the first and second parcels had 6 items, the third parcel had 4 items.

### 5.4. Plan of Analysis

We conducted a three-wave longitudinal design, with one month between each wave among a large sample of Chilean workers (*N* = 735). We tested three different CLPMs. Model 1 tested the prospective link between OCBs and hedonic happiness. Model 2 tested the prospective link between OCBs and eudaimonic happiness. Model 3 tested the prospective link between OCBs and flourishing. For each CLPM, each measure at (T + 1) was regressed on its own lagged measure at (T) as well as on the other lagged measure at (T) [88]. We allowed OCBs and each measure of happiness to covary freely within each time point. Thus, all constructs were represented as potential antecedents and potential consequences of the other construct, while controlling for stability paths.

Our sample size and the three-wave design allowed us to use rigorous statistical analyses. Modeling latent variables reduces the biasing effects of measurement error [88], providing stronger estimates of stability paths and thus more stringent tests of the hypothesized cross-lagged parameters. We used MPlus 7.1 [89] to model the relations among the latent variables. Descriptive statistics and correlations are shown in Table 2 (Model 1), Table 3 (Model 2), and Table 4 (Model 3). Following the recommendations of Hu and Bentler [90] and Kline [80], we assessed the model fit through the root mean square error of approximation (RMSEA) and comparative fit index (CFI), with values of RMSEA < 0.06 (0.08) and CFI > 0.95 (0.90) indicating acceptable fit.

## 6. Results

### 6.1. Model 1: The Longitudinal Link between OCBs and Hedonic Happiness

**Figure 1:**
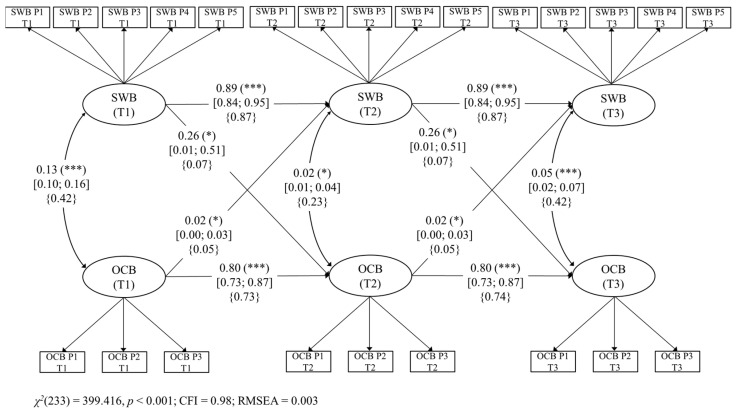
Model 1. Structural longitudinal model for the associations between subjective well-being (SWB) and organizational citizenship behaviors (OCBs). Coefficients shown are unstandardized paths. The unstandardized confidence intervals are reported in square brackets for significant paths. Standardized paths are shown in curly parentheses. Error terms and factor loadings are not shown to enhance visual clarity. P1: Parcel 1, P2: Parcel 2, P3: Parcel 3, P4: Parcel 4, and P5: Parcel 5. T1: Time 1, T2: Time 2, and T3: Time 3. Solid lines = significant paths. *** *p* < 0.001; * *p* < 0.05.

**Figure 2:**
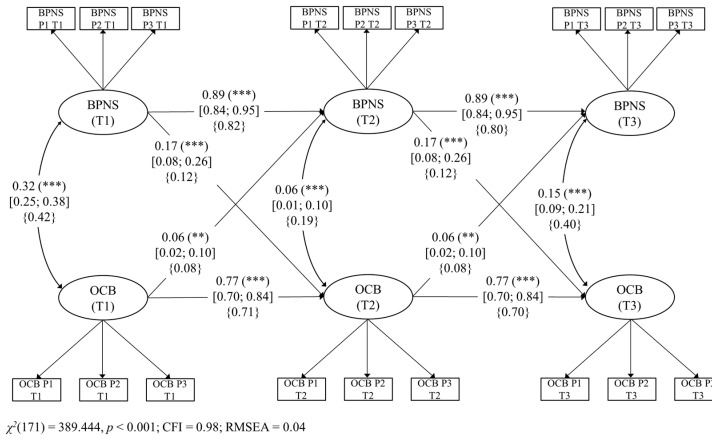
Model 2. Structural longitudinal model for the associations between basic psychological need satisfaction (BPNS) and organizational citizenship behaviors (OCBs). Coefficients shown are unstandardized paths. The unstandardized confidence intervals are reported in square brackets for significant paths. Standardized paths are shown in curly parentheses. Error terms and loadings are not shown to enhance visual clarity. P1: Parcel 1, P2: Parcel 2, P3: Parcel 3, and P4: Parcel 4. T1: Time 1, T2: Time 2, and T3: Time 3. Solid lines = significant paths. *** *p* < 0.001; ** *p* < 0.01.

**Figure 3:**
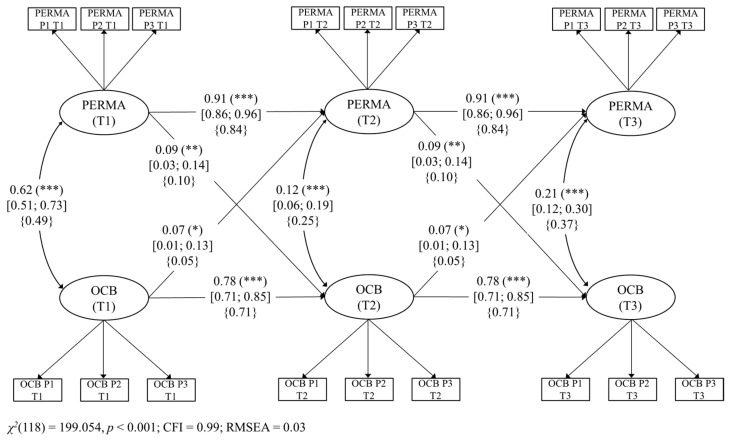
Model 3. Structural longitudinal model for the associations between PERMA and organizational citizenship behaviors (OCBs). Coefficients shown are unstandardized paths. The unstandardized confidence intervals are reported in square brackets for significant paths. Standardized paths are shown in curly parentheses. Error terms and loadings are not shown to enhance visual clarity. P1: Parcel 1, P2: Parcel 2, and P3: Parcel 3. T1: Time 1, T2: Time 2, and T3: Time 3. Solid lines = significant paths. *** *p* < 0.001; ** *p* < 0.01; * *p* < 0.05.

First, we started with a CLPM without any constraints. This model fit the data well, χ^2^(217) = 384.236, *p* < 0.001, CFI = 0.977, and RMSEA = 0.032 (90% CI: 0.027, 0.038). Second, we constrained all the factor loadings of each latent variable to be equal across waves. The model also fit the data well, χ^2^(229) = 396.916, *p* < 0.001, CFI = 0.977, and RMSEA = 0.032 (90% CI: 0.026, 0.037). According to Cheung and Rensvold [91], the assumption of invariance is tenable if the reduction in CFI, when constraints are imposed, is less than 0.01. Here, the change in CFI met this criterion (ΔCFI = 0.000). Thus, we considered it acceptable to assume invariance for this CLPM and kept this constraint further. Third, and finally, we made the simplifying assumption of constraining autoregressive and cross-lagged paths to be invariant over time in our last model [92]. In other words, we assumed that “the paths do not differ across the time points (i.e., a stationary process; [93])”, as recommended by Olafsen et al. [94] (p. 280). Indeed, the time distance between T1–T2 is the same time distance between T2–T3 (4 weeks), and we do not have any conceptual or theoretical reason to assume that the paths would differ between waves. Because of that, the interplay of variables would be in a stationary state, implying that every predictive path between pairs of waves is conceptually equivalent, which allows us to build collapsed and more parsimonious models [94] as well as gain statistical power. Hence, each hypothesis was represented by a single parameter representing the combined effect from T1 to T2 and from T2 to T3 (we maintained this assumption for Model 2 and Model 3 below). This final model showed a good fit, χ^2^(233) = 399.416, *p* < 0.001, CFI = 0.977, and RMSEA = 0.031 (90% CI: 0.026, 0.036). Unstandardized factor loadings ranged from 0.941 to 1.496 (all *p* < 0.001). For simplicity, because unstandardized paths between T1–T2 are the same as paths between T2–T3, we only reported the former (T1–T2) within the text. Structural parameters for our CLPM are reported in detail in Figure 1. We followed the same procedure in Model 2 (Figure 2) and Model 3 (Figure 3). Supporting H1a, hedonic happiness at T1 was a positive prospective predictor of OCBs at T2 (β = 0.26, [95% CI 0.007, 0.506], *p* = 0.04). Supporting H1b, OCBs at T1 was a significant positive prospective predictor of hedonic happiness at T2 (β = 0.02, [95% CI. 0.00, 0.032], *p* = 0.04).

### 6.2. Model 2: The Longitudinal Link between OCBs and Eudaimonic Happiness

First, we started with a CLPM without any constraints. This model fit the data well, χ^2^(157) = 362.388, *p* < 0.001, CFI = 0.981, RMSEA = 0.042 (90% CI: 0.037, 0.048). Second, we constrained all the factor loadings of each latent variable to be equal across waves. The model also fit the data well, χ^2^(167) = 379.039, *p* < 0.001, CFI = 0.981, RMSEA = 0.042 (90% CI: 0.036, 0.047). Because the reduction in CFI is less than 0.01 (ΔCFI = 0.000), we considered it acceptable to assume invariance for this CLPM and kept this constrained further [91]. Third, and finally, we constrained autoregressive and cross-lagged paths to be invariant over time [92,94]. This final model showed a good fit, χ^2^(171) = 389.444, *p* < 0.001, CFI = 0.980, and RMSEA = 0.042 (90% CI: 0.036, 0.047). Unstandardized factor loadings ranged from 0.941 to 1.085 (all *p* < 0.001). Structural parameters for our CLPM are reported in detail in Figure 2. Supporting H2a, eudaimonic happiness at T1 was a positive prospective predictor of OCBs at T2 (β = 0.17, [95% CI 0.080, 0.260], *p* < 0.001). Supporting H2b, OCBs at T1 were significant positive prospective predictors of eudaimonic happiness at T2 (β = 0.06, [95% CI. 0.023, 0.103], *p* = 0.002).

### 6.3. Model 3: The Longitudinal Link between OCBs and Flourishing

First, we started with a CLPM without any constraints. This model fit the data well, χ^2^(106) = 184.076, *p* < 0.001, CFI = 0.992, and RMSEA = 0.032 (90% CI: 0.024, 0.039). Second, we constrained all the factor loadings of each latent variable to be equal across waves. The model also fit the data well, χ^2^(114) = 192.931, *p* < 0.001, CFI = 0.992, and RMSEA = 0.031 (90% CI: 0.023, 0.038). Because the reduction in CFI is less than 0.01 (ΔCFI = 0.000), we considered it acceptable to assume invariance for this CLPM and kept this constraint further [91]. Third, and finally, we constrained autoregressive and cross-lagged paths to be invariant over time [92,94]. This final model showed a good fit, χ^2^(118) = 199.054, *p* < 0.001, CFI = 0.991, and RMSEA = 0.031 (90% CI: 0.023, 0.038). Unstandardized factor loadings ranged from 0.940 to 1.095 (all *p* < 0.001). Structural parameters for our CLPM are reported in detail in Figure 3. Supporting H3a, flourishing at T1 was also a positive prospective predictor of OCBs (β = 0.09, [95% CI 0.034, 0.140], *p* = 0.001). Supporting H3b, OCBs at T1 was a significant positive prospective predictor of flourishing at T2 (β = 0.07, [95% CI 0.006, 0.134], *p* = 0.03).

## 7. Discussion

Although a few studies had made attempts to explore the link between OCBs and happiness in the organizational context before our research, there were still important gaps that needed to be addressed. First, the scarcity of studies on the link between OCBs and happiness have only tested individual facets of their respective models (e.g., affect, relatedness, meaning) instead of testing comprehensive models of happiness as a whole construct (e.g., hedonic happiness, eudaimonic happiness, and flourishing). Second, the directionality of the link is still unknown. Most research has assumed that happiness is the driver of OCB. However, we hypothesized that the link would be reciprocal. Third, most previous research was conducted in the Western World with a few exceptions in Asia. Thus, more research is needed in different cultural backgrounds, such as Latin-American. In response to the identified research gaps, we conducted a three-wave longitudinal design in order to test the prospective link between OCBs and each of the three mentioned models of happiness among Chilean workers in an organizational context. Key findings emerge from our research. First, we found that OCBs are prospective positive predictors of hedonic happiness, eudaimonic happiness, and flourishing. Second, the three models of happiness prospectively predict higher OCBs. Thus, we showed, for the first time, that OCBs and each of the three models of happiness are reciprocally linked in a virtuous circle of prosociality and well-being in the workplace.

In spite of the significant reciprocal links found between OCBs and the three models of happiness, it is worth mentioning that the strengths of the relationships seem to be different in all of them. Unfortunately, since we ran three different models, our design does not allow the analysis of statistical differences between the respective paths across Model 1, Model 2, and Model 3. We are only able to comment on these differences as tendencies. It is relevant to notice that, in order to compare the strength of the effect sizes, we are focusing on the standardized paths reported in Figure 1, Figure 2 and Figure 3 (curly parentheses). For example, regarding the link from happiness to OCBs, our results show that the stronger predictor of OCBs seems to be BPNS (eudaimonic happiness; *β* = 0.12), followed by PERMA (flourishing; *β* = 0.10) and then by SWB (hedonic happiness; *β* = 0.07). Although we did not hypothesize these differences, these preliminary results could make theoretical sense. Indeed, following SDT (Ryan & Deci, 2017), the satisfaction of the needs for autonomy, competence, and relatedness (i.e., BPNS) is one of the most powerful motivational forces, giving workers the vital energy they need to engage in behaviors such as helping colleagues at work [68].

Regarding the reverse link from OCBs to happiness, our results show that OCBs seem to predict more strongly BPNS (eudaimonic happiness; *β* = 0.08), then PERMA (flourishing; *β* = 0.05), and finally SWB (hedonic happiness; *β* = 0.05). These results tend to show that OCBs predict more strongly eudaimonic than hedonic happiness. In fact, we think that it might be arguable that the act of helping others is more related to the actualization of human potentials, meaning, and self-realization (i.e., eudaimonia) than to the search for pleasure and joy (i.e., hedonia).

These tendencies are theoretically interesting; however, they would need to be tested more rigorously. They also open a new set of questions. For example, which conceptualization of happiness better predicts OCBs and why? Do OCBs have a stronger effect on hedonic or eudaimonic components of happiness? Future research may approach these questions using more sophisticated models and analyses.

### 7.1. Theoretical and Practical Implications

The present research aims to extend the field of POS theoretically and practically by bringing new insights to academics and practitioners in this sphere. Indeed, we expect to contribute to the improvement of workers’ quality of life, as well as building healthier and more resilient organizations [20], through a better understanding of the relationship between happiness and prosociality within the workplace.

#### 7.1.1. Theoretical Implications

Our results extend the study of the OCBs–happiness link in five important ways. First, we conceptualized happiness from a broader perspective than in previous research in the field. By highlighting that happiness is a broad construct, we are promoting insights to scholars interested in deepening the knowledge of the link between OCBs and happiness. This could encourage the collaboration between OCBs and happiness scholars. We see a tremendous potential for the mutual contribution of these two research areas that can inform each other and go beyond the mere emotional aspects of well-being in the workplace.

Second, happiness has been conceptualized as either an antecedent or a consequence of OCBs. We tested the OCBs–happiness link and demonstrated for the first time that it is reciprocal for all happiness “conceptualizations”. Third, we extended previous research mainly conducted in the Western world and Asia, by testing our hypotheses with data from a Chilean sample. Chile presents several cultural aspects that differ from the countries previously studied, hence contributing to the cross-cultural generalization of previous findings. Fourth, as Bolino and Grant have stated [95] “one natural direction for future research is to develop a comprehensive model of prosociality in organizations” (p. 647). By studying the relationship between happiness and OCBs in more detail, we are contributing in the direction of a more integrated theoretical approach to studying prosociality. Because we showed that both constructs are mutually interconnected, it is difficult to think in a comprehensive model of OCBs without including the role of happiness.

Fifth, and finally, it has been suggested to keep inquiring about possible antecedents and consequences of OCBs [5]. Indeed, it is extremely important to include factors that have raised attention in the 21st century but have not “been comprehensively explored and understood well in the current literature” of OCBs [5]. Some factors that were historically ignored have been gaining more attention and relevance (e.g., RSE), and happiness is an example. In fact, the study of the OCBs–happiness link is nascent [95], and its association with OCBs deserves more attention. Further, our results contribute significantly to the OCBs’ nomological network: happiness appears to be not only a cause, but also a consequence of OCBs.

#### 7.1.2. Practical Implications

Three practical contributions emerge from our research. First, our results strongly encourage workers and organizations to attach higher importance to kindness, generosity, and prosociality in the workplace (e.g., OCBs) in order to foster employees’ happiness, but also companies’ sustainability and profitability. In other words, “doing good feels good”. Importantly, we provided robust evidence that performing OCBs not only increases hedonic happiness, but also eudaimonic happiness and flourishing.

Second, we also encourage employees and companies to attach high importance to happiness in the work context. We showed that increments in any of the three conceptualizations of happiness lead to higher OCBs, which in turn are associated not only with workers’ wellness, but also with more desirable organizational outcomes (e.g., productivity, customer satisfaction, job satisfaction, trust, etc.).

Third, companies and practitioners count now on a broader set of tools for fostering OCBs through, for example, happiness programs. Our results show that intervention programs are not restricted only to promoting positive emotions/affect (hedonic happiness) in order to make OCBs more likely. In fact, practitioners could also expand their interventions toward increasing psychological needs satisfaction (eudaimonic happiness) as well as toward meaning and engagement (key components of the flourishing perspective) in order to increase the consequent appearance of OCBs.

### 7.2. Limitations and Further Research

Some limitations of the current research must be noted. First, our measures were self-reported, and shared method variance could potentially have inflated correlations among constructs within each wave. However, self-reports of one’s experience are arguably the most valid ways of measuring happiness. Additionally, a recent meta-analysis showed that mean differences between self- and other- ratings of OCBs are quite small [96]. Importantly, we also took several a priori precautions to mitigate common-method bias, such as using construct-valid measurement scales, protecting respondent anonymity, and instructing the participants that there were no right or wrong answers [97,98].

Second, although the prospective effects reported here through our CLPM substantially strengthen the case for causal effects between OCBs and happiness, by providing evidence of temporal precedence, they do not provide conclusive evidence for causality. For example, unmeasured variables may be at play at work. Nonetheless, our longitudinal design strongly improved our knowledge regarding the temporal link between OCBs–happiness. Third, the pattern of results obtained may have been affected by the time-lag chosen between waves. We tested a three-wave CLPM with one month between waves. Future research should explore different time-lags and use intensive longitudinal data analysis. Fourth, caution is needed about generalizing the findings from a Chilean sample to other samples and research contexts. Further research needs to expand our findings to other economic, social, and cultural contexts. Fifth, we only tested the direct link between OCBs and happiness. It would be interesting for future research to assess mediators and moderators. Sixth, although we found significant reciprocal links between OCBs and the three models of happiness, the effect sizes were small (see [99]). Nonetheless, it is a standard issue when using CLPM. Indeed, in a standard CLPM, all constructs are controlled by their stability path, which reduces significantly the variance explained for the construct itself.

Finally, as stated in the discussion, the potential different effect sizes for the link between the three conceptualizations of happiness and OCBs (in both directions) could only be discussed in terms of tendencies in our study. From a theoretical and a practical perspective, it would be very informative to know if the differences observed here are stable in future studies. We would not like to jump to the conclusion that the eudaimonic perspective is a stronger predictor of OCBs until further evidence is found. Future research should include designs that more specifically search for these differences in effect sizes.

Despite these limitations, we believe our research strengthens the knowledge regarding the OCBs–happiness link by conducting an original longitudinal design and demonstrating that the inclusion of the three conceptualizations of happiness makes our understanding of this relationship richer and worthy of being pursued in future research endeavors.

## 8. Conclusions

The present research shows that, in the workplace, “doing good feels good” and “feeling good leads to doing good”. Importantly, “doing good” (i.e., OCBs) leads to being happier in terms of all its three conceptualizations: hedonic, eudaimonic, and flourishing. Additionally, “feeling good” in any sense (i.e., any kind of happiness) will foster “doing good” (i.e., OCBs). Following this wordplay, it does not matter if you are “feeling good” because of high positive emotions, high autonomy, or having more meaning in your life. Any kind of happiness on its own, considering any of the psychological approaches covered in this research, will favor prosocial behaviors in the workplace. This powerful virtuous circle of prosociality and happiness might benefit not only organizations’ profitability, but also the well-being and happiness of the “good soldiers” [6].

## Figures and Tables

**Table 1:** Participant’s profile who answered T1 (*N* = 735).

Variables	Categories	Gender							
		Total		Male		Female		Other	
		*N*	%	*N*	%	*N*	%	*N*	%
	Sample	735		418		315		2	
Age range	18–25	40	5.4	14	3.3	26	8.3	0	0.0
	26–45	520	70.7	281	67.2	237	75.2	2	100.0
	46–55	128	17.4	81	19.4	47	14.9	0	0.0
	56 or more	47	6.4	42	10.0	5	1.6	0	0.0
Education	High school	11	1.5	7	1.7	3	1.0	1	50.0
	Incomplete college education	71	9.7	44	10.5	27	8.6	0	0.0
	Bachelor’s Degree	373	50.7	200	47.8	172	54.6	1	50.0
	Postgraduate	250	34.0	154	36.8	96	30.5	0	0.0
	Other	30	4.1	13	3.1	17	5.4	0	0.0
Marital status	Single	262	35.6	121	28.9	140	44.4	1	50.0
	Married	301	41.0	212	50.7	89	28.3	0	0.0
	Divorced	90	12.2	42	10.0	47	14.9	1	50.0
	Cohabited	77	10.5	42	10.0	35	11.1	0	0.0
	Widow/widower	1	0.1	0	0.0	1	0.3	0	0.0
	Other	4	0.5	1	0.2	3	1.0	0	0.0
Activity	Marketing	36	4.9	22	5.3	14	4.4	0	0.0
	Finance	78	10.6	47	11.2	31	9.8	0	0.0
	Accounting	46	6.3	24	5.7	22	7.0	0	0.0
	Operations	129	17.6	85	20.3	42	13.3	2	100.0
	Technology	70	9.5	26	6.2	44	14.0	0	0.0
	People/Human Resources	55	7.5	43	10.3	12	3.8	0	0.0
	Administrative	99	13.5	49	11.7	50	15.9	0	0.0
	Other	222	30.2	122	29.2	100	31.7	0	0.0
Leadership role	Yes	478	65.0	300	71.8	177	56.2	1	50.0
	No	257	35.0	118	28.2	138	43.8	1	50.0

**Table 2:** Descriptive and inter-correlations for Model 1 variables.

	M	SD	1	2	3	4	5	6	7	8	9	10	11	12	13
1. Gender															
2. Age	38.77	9.69	−0.20 **												
3. Positive Affect T1	4.01	0.58	−0.08 *	0.16 **											
4. Positive Affect T2	3.99	0.59	−0.11	0.14 *	0.70 **										
5. Positive Affect T3	3.92	0.61	−0.14 *	0.18 **	0.66 **	0.74 **									
6. Negative Affect T1	2.62	0.61	0.10 **	−0.20 **	−0.18 **	−0.21 **	−0.16 **								
7. Negative Affect T2	2.56	0.64	0.15 **	−0.19 **	−0.25 **	−0.23 **	−0.19 **	0.79 **							
8. Negative Affect T3	2.53	0.65	0.10	−0.13 *	−0.28 **	−0.20 **	−0.28 **	0.71 **	0.76 **						
9. Life Satisfaction T1	4.47	1.05	−0.03	0.07	0.37 **	0.39 **	0.39 **	−0.42 **	−0.48 **	−0.45 **					
10. Life Satisfaction T2	4.56	1.14	−0.11	0.14 *	0.37 **	0.42 **	0.49 **	−0.46 **	−0.53 **	−0.51 **	0.87 **				
11. Life Satisfaction T3	4.50	1.12	−0.05	0.08	0.43 **	0.42 **	0.50 **	−0.44 **	−0.49 **	−0.54 **	0.83 **	0.89 **			
12. OCB T1	4.68	1.01	−0.03	0.17 **	0.38 **	0.39 **	0.44 **	−0.21 **	−0.23 **	−0.28 **	0.36 **	0.37 **	0.44 **		
13. OCB T2	5.65	1.07	−0.12 *	0.18 **	0.35 **	0.41 **	0.44 **	−0.26 **	−0.31 **	−0.30 **	0.42 **	0.44 **	0.44 **	0.70 **	
14. OCB T3	4.46	1.27	−0.20 **	0.23 **	0.34 **	0.33 **	0.53 **	−0.21 **	−0.30 **	−0.32 **	0.36 **	0.42 **	0.46 **	0.62 **	0.75 **

OCB: organizational citizenship behavior. ** *p* < 0.01; * *p* < 0.05.

**Table 3:** Descriptive and inter-correlations for Model 2 variables.

	M	SD	1	2	3	4	5	6	7
1. Gender									
2. Age	38.77	9.68	−0.20 **						
3. BPNS T1	5.91	0.80	−0.06	0.07					
4. BPNS T2	5.89	0.90	−0.15 **	0.16 **	0.85 **				
5. BPNS T3	5.78	0.98	−0.12 *	0.13 *	0.78 **	0.84 **			
6. OCB T1	4.68	1.01	−0.03	0.17 **	0.41 **	0.41 **	0.48 **		
7. OCB T2	5.65	1.07	−0.12 *	0.18 **	0.46 **	0.49 **	0.53 **	0.70 **	
8. OCB T3	4.46	1.27	−0.20 **	0.23 **	0.45 **	0.38 **	0.56 **	0.62 **	0.75 **

BPNS: basic psychological need satisfaction. OCB: organizational citizenship behavior. ** *p* < 0.01; * *p* < 0.05.

**Table 4:** Descriptive and inter-correlations for Model 3 variables.

	M	SD	1	2	3	4	5	6	7
1. Gender									
2. Age	38.77	9.69	−0.20 **						
3. PERMA T1	7.53	1.37	−0.10 **	0.10 **					
4. PERMA T2	7.63	1.53	−0.15 **	0.13 *	0.84 **				
5. PERMA T3	7.69	1.59	−0.11	0.11 *	0.81 **	0.85 **			
6. OCB T1	4.68	1.01	−0.03	0.17 **	0.45 **	0.47 **	0.46 **		
7. OCB T2	5.65	1.07	−0.12 *	0.18 **	0.46 **	0.52 **	0.50 **	0.70 **	
8. OCB T3	4.46	1.27	−0.20 **	0.23 **	0.46 **	0.45 **	0.55 **	0.62 **	0.75 **

OCB: organizational citizenship behavior. ** *p* < 0.01; * *p* < 0.05.

## Data Availability

Not applicable.

## Abbreviations

BPNS	Basic psychological needs
BPNT	Basic psychological needs theory
CFI	Comparative fit index
CLPM	Cross-lagged panel models
OCBs-I	Prosocial behaviors towards individuals
OCBs-O	Prosocial behaviors towards the organization
OCBs	Organizational citizenship behaviors
POS	Positive Organization Psychology
PSBs	Prosocial behaviors
Hi	Hypothesis Hi
RMSEA	Root mean square error of approximation
SDT	Self-determination theory
PERMA	Seligman (2011) model of flourishing
SEM	Structural equation modeling
SWB	Subjective well-being
